# The Impact of Foreign Language on Meta-Reasoning in Moral Decisions

**DOI:** 10.5334/joc.472

**Published:** 2026-01-07

**Authors:** Zhimin Hu, Beatriz Martín-Luengo, Eduardo Navarrete

**Affiliations:** 1 Department of Developmental Psychology and Socialisation, University of Padua, Italy; 2 Department of Language Science and Technology, The Hong Kong Polytechnic University, Hong Kong; 3 Center for Cognition and Decision Making, Institute for Cognitive Neuroscience, HSE University, Moscow, Russia; 4 Departamento de Psicología y Sociología, Facultad de Ciencias Sociales y Humanas, University of Zaragoza, Spain

**Keywords:** foreign language effect, metacognition, feeling of rightness, bilingualism, moral dilemmas

## Abstract

This research investigates the moral Foreign Language Effect (mFLE) from a metacognitive perspective. Grounded in the Dual-Process framework, previous research posits using a foreign language evokes more utilitarianism by dampening emotional responses and promoting analytical reasoning. However, the role of metacognition remains underexplored. The study hypothesizes that reasoning in a foreign language will lower the Feeling of Rightness (FOR), reflecting increased uncertainty and prompting more reevaluation. Adopting a decision-redecision paradigm, participants’ responses to moral dilemmas in their native and foreign languages were compared. Analytical methods included linear mixed effects models to evaluate language effects on decisions, decision times, redecision times, FORs, Final Judgment of Confidence (FJC), and decision reversals, with language proficiency considered as a potential moderating factor. Across two preregistered studies, results indicated that while there was no FLE in moral decisions or inclinations, foreign language impacted metacognition. Study 1 found that foreign language significantly increased decision times while lowering FOR and FJC with a higher rate of decision reversals. Study 2, using a Process Dissociation approach, revealed a nuanced understanding of FLE on metacognition in relation to relative proficiency and specific dilemma sets. Across both studies, lower FOR was consistently correlated with longer redecision times and a higher probability of decision reversal, confirming its role in prompting analytical thinking. The findings aim to further enhance the understanding of the FLE, providing insight on how language might alter metacognitive monitoring and control. This research holds implications for decision-making in multilingual contexts, emphasizing language’s role in cognitive and metacognitive processes.

## 1. Introduction

Imagine being faced with a difficult moral dilemma: Sacrificing the life of one person to save the lives of five other people. Would your decision vary if you were reasoning in your native language compared to a foreign language? Studies have indicated that the language we use during decision-making can indeed influence the outcomes of such moral decisions (Costa et al., 2014b; Geipel et al., 2015a). But if you were to reflect on the rightness of your decision, would the language in which the decision was made affect your introspection? The Foreign Language Effect (FLE), conceptualized by Keysar, Hayakawa, and An (2012), describes the phenomenon when reasoning in a foreign language individuals exhibit less cognitive biases during judgments and decision-making compared to in their native language. Several hypotheses have endeavored to explain the FLE under the Dual-Process framework (Evans, 2006; Kahneman, 2003; Sloman, 2002), suggesting that FLE could arise from increased deliberation, reduced emotions, or a combination of both when reasoning in a foreign language (Chan et al., 2016; Hayakawa et al., 2017). However, while these hypotheses help elucidate the cognitive and affective aspects of the FLE, the metacognitive aspect of human judgment and decision-making has seldom been discussed. Metacognition, or thinking about one’s own thinking, plays a decisive role in judgment and decision making. An example of metacognitive processes would be that we often pause to evaluate the appropriateness of our immediate responses before providing our final answers. Concerning the underlying mechanisms of the FLE, an important question that arises is: Other than affective and cognitive processes, does a foreign language also impact metacognitive processes? In this pre-registered study, we aim to examine the FLE from a metacognitive perspective. Further understanding of the FLE has important social implications in an ever more multilingual world, as languages could be used as a tool to nudge people’s behaviors either for better (Geipel et al., 2022; Nicolao et al., 2018) or for worse (Muda et al., 2023; Pavlenko, 2017). Thus, a comprehensive understanding of underlying mechanisms of the FLE by including the metacognitive aspect is strongly warranted.

### 1.1 Foreign language effect

In the foundational study by Keysar et al. (2012), it was notably observed that participants displayed reduced susceptibility to the framing effect during decision-making tasks in their foreign language compared to their native language. This decision task, which finds its roots in the work of Tversky and Kahneman (1981), places individuals in a scenario, where a fictitious disease is expected to kill 600 individuals. Participants have to choose between two alternative programs with identical outcomes to combat the disease. The first program (a risk-averse option) will save 200 people for sure, while the second program (a risk-seeking option) has a 1/3 probability of saving everyone and a 2/3 probability of saving no one. The framing effect is when participants presented with the positive frame (lives saved) tend to prefer the safe option compared to the risky option; while this preference is reduced or disappears in those that are presented with the negative frame (lives lost). In Keysar et al.’s (2012) study, it was found that participants were less influenced by the framing of the options when the decision task was in a foreign language. These authors argued that individuals tend to make more rational, less emotionally-driven decisions when thinking in a foreign language as opposed to their native tongue. These results have sparkled a burgeoning line of research in the FLE that has corroborated and extended these findings, demonstrating a reduction of several cognitive biases when thinking in a foreign language as compared to their native one, such as risk aversion (Costa et al., 2014a; Hayakawa et al., 2019), hot-hand fallacy (Gao et al., 2015), causality bias (Díaz-Lago & Matute, 2019b), and endowment effect (Karataş, 2020) amongst others. In particular, the FLE has been extensively investigated in the realm of moral psychology, with evidence demonstrating that individuals exhibit more utilitarian tendencies when moral dilemmas, particularly the Footbridge-like dilemmas, are presented in a foreign language compared to their native language (Cipolletti et al., 2016; Corey et al., 2017; Geipel et al., 2015a, 2015b; Miozzo et al., 2020; Muda et al., 2020a; Wong & Ng, 2018). Recent meta-analyses have shown a small yet robust effect (Cipolletti et al., 2016; Del Maschio et al., 2022; Stankovic et al., 2022).

It is worth noting that the boundaries of the FLE are not always clear. For example, there are several studies have reported no differences in the outcome bias (Vives et al., 2018) and gambling-related biases (Muda et al., 2020b) between native and foreign languages, while others have reported increased susceptibility to biases such as acquiescence (Hu et al., 2024), the Moses illusion (Dhaene et al., 2022) and fake news (Muda et al., 2023) in a foreign language. Potential factors contributing to these divergent results in FLE relate to individual differences, such as the proficiency in the foreign language (Del Maschio et al., 2022) and the similarity between the tested languages, e.g., Swedish-Norwegian (Dylman & Champoux-Larsson, 2020). Nevertheless, the majority of the literature on the FLE has reported a positive effect, particularly in the moral domain shown by multiple meta-analyses (Cipolletti et al., 2016; Corey et al., 2017; Del Maschio et al., 2022; Hayakawa et al., 2017; Stankovic et al., 2022).

### 1.2 Mechanisms of the FLE

Central to the FLE is the application of the Dual-Process framework (Kahneman, 2003). This framework delineates two distinct systems of processing: System 1 and System 2. System 1 encapsulates a fast, intuitive, automatic, nonvoluntary, and affect-driven processing style. It is often associated with heuristic thinking, whereby decisions are made swiftly, albeit sometimes impulsively. On the contrary, System 2 embodies a slow, deliberate, systematic, voluntary, and cognitively controlled processing mechanism. This system is linked with deliberative reasoning, where decisions are carefully evaluated and pondered upon before being made (Kahneman & Frederick, 2002; Sloman, 1996). When applied to moral reasoning, the Dual-Process framework elucidates the contrasting deontological and utilitarian inclinations that individuals may harbor in order to ascertain the most beneficial outcome, even if it entails causing harm. There are at least two interpretations of the interaction of language and moral decision. One view states that using a foreign language reduces the impact of intuitive emotional reasoning associated with System 1, blunting deontology (Costa et al., 2014b; Hayakawa et al., 2017). The other interpretation states that foreign language processing activates systematic processing, characteristic of System 2, promoting more utilitarian tendencies (Chan et al., 2016; Cipolletti et al., 2016). However, both interpretations point to the same outcome, that is, the triumph of utilitarianism over deontology in a foreign language. We detail below these two main hypotheses.

The *reduced emotion hypothesis* posits that this reduction in emotional resonance in a foreign language could curb the automatic, affect-based responses to harmful actions engendered by System 1, as people typically feel emotionally more distant for concepts and ideas in language that is less dominant and less fluent (Dewaele, 2008; Kazanas et al., 2019). Consequently, the diminished influence of System 1 paves the way for the emergence of more analytical System 2, giving rise to more utilitarian decisions (Costa et al., 2014b). This hypothesis aligns well with copious psychophysiological evidence in second language processing research. For example, emotions, particularly the negative ones, are less charged in a foreign language compared to a native language, showcased by less sensitivity in skin conductance (Caldwell-Harris et al., 2011; Caldwell-Harris & Ayçiçeği-Dinn, 2009), pupil dilation (Duñabeitia & Costa, 2015; García-Palacios et al., 2018), electroencephalographic signals (Gao et al., 2015), and motor movements (Incera et al., 2020). Further supporting this hypothesis are the findings that demonstrate detached moral norms (Geipel et al., 2015b) and social norms (Gawinkowska et al., 2013; Hu & Navarrete, 2024), thus rendering the emotions felt in violating such norms less salient.

The *increased deliberation hypothesis* posits that the need to process information in a cognitively more challenging context induces more deliberative and cognitively controlled thinking style, leading people to prefer System 2 over System 1 in decision-making tasks in a foreign language (Keysar et al., 2012). This hypothesis is grounded on the observation that processing a foreign language, whether in text or speech, entails reduced cognitive fluency (Abutalebi, 2008; Segalowitz, 2010; Volk et al., 2014). The level of processing fluency can influence the cognitive processes engaged in task resolution as decreased processing fluency often triggers deliberative and analytical processes (Alter et al., 2007; Oppenheimer, 2008). For example, researchers have observed enhanced performance in reasoning tasks when information is rendered in a disfluent font (Alter et al., 2007), though it is worth mentioning that such an effect has not been replicated in math problems (Meyer et al., 2015), potentially due to different cognitive processes involved in math problems and reasoning tasks. Nonetheless, in tasks with decreased processing fluency, researchers have consistently observed a diminution of cognitive biases such as the Moses illusion (Song & Schwarz, 2008), belief in conspiracy theories (Swami et al., 2014), and causality bias (Díaz-Lago & Matute, 2019a, but see, Bona & Vicovaro, 2024). Based on these theoretical considerations, several researchers have manipulated the language of experimental tasks as a function of processing fluency, reporting a reduction in cognitive bias such as framing effects (Huang & Rau, 2020; Keysar et al., 2012) and causality bias (Díaz-Lago & Matute, 2019b).

These two hypotheses provide valuable insight into the affective and cognitive mechanisms of the FLE. In other words, when thinking in a foreign language people would be less swayed by the intuitive emotion-driven System 1 and more likely to tune in on the analysis-focused System 2, leading to less deontological but more utilitarian tendencies. However, these two hypotheses have also faced criticisms. For example, McFarlane and Cipolletti Perez (2020) challenge the validity of the *reduced emotion hypothesis* for using proxies for emotions, lacking generalizable emotion types, and not controlling for baseline emotional levels. In fact, people often experience negative emotions such as anxiety when using a foreign language (MacIntyre & Gardner, 1994; Naser Oteir & Nijr Al-Otaibi, 2019; Woodrow, 2006). Thus, although some negative emotions might be attenuated when thinking in a foreign language, there might be others evoked by merely having to think in a foreign language. Furthermore, Miozzo and colleagues (2020) have extended the preference for utilitarian responses to regional languages. In contrast to foreign languages, regional languages are acquired in infancy with comparable proficiency levels of the national language. Specifically, Miozzo and colleagues (2020) showed emotional responses are equally intense in the regional languages (e.g., Venetian) and the national language (i.e., Italian), arguing that rather than a generalized emotional reduction, it is the context in which the language is acquired that affects their decisions. Therefore, it is challenging to conclude a generalized emotional reduction to account for the FLE. As for the *increased deliberation hypothesis*, if it holds, one would also expect to observe better performance in the cognitive reflection test (CRT) in a foreign language compared to a native language. However, previous research has not revealed any differences in CRT performance in native and foreign languages (Costa et al., 2014a; Vega-Mendoza et al., 2021), bringing the validity of this hypothesis under scrutiny. Thus, an important question to ask is do these two hypotheses sufficiently account for the FLE? For instance, Hadjichristidis et al. (2017) pointed out that differences in memory processes between native and foreign languages using the brain-drain model could be held responsible for the FLE. Thus, given the complexity of such a phenomenon and often mixed results, Polonioli (2018) has called for alternative explanations for the FLE. In sum, although the *reduced emotions hypothesis* and the *increased deliberation hypotheses* explain the FLE to a certain extent, they may not be sufficient in explaining the FLE in its complexity.

A crucial aspect unaccounted for by existing hypotheses based on the dual-process account concerns how individuals “switch” from the more dominant System 1 to less intuitive System 2. In other words, do emotional distance and decreased fluency directly deactivate and activate System 1 and System 2, respectively, or are their impacts on reasoning via another mechanism? To answer this question, an inquiry through the lens of metacognition is needed, because the activation of System 2 reasoning to a large extent depends on one’s metacognitive experiences in reasoning (Fletcher & Carruthers, 2012; Lieder & Griffiths, 2017). In the FLE literature, there is only a limited number of studies that glossed over this aspect. For instance, Białek et al (2020) reported that people made less logical evaluations in a foreign language than in their native language, attributing this result to lessened “metacognitive tracking” ability in the foreign language. Similarly, Mækelæ & Pfuhl (2019) showed that a foreign language did not directly increase deliberate thinking per se, but rather it increased people’s willingness to spend more cognitive effort and time to process the task. Most recently, one study found that people seemed to be better at monitoring their memory in a foreign language than in a native language (Grant et al., 2023). These results mirror Circi et al.’s (2021) discussion on a sort of “cognitive control mechanism” that could underpin the FLE. Albeit not having directly linked the FLE to metacognition, the terminologies used in these studies, such as metacognitive tracking, allocation of cognitive resources, monitoring, and cognitive control are all key concepts in metacognition. In fact, several authors have alluded to the potential distortions of metacognitive processes when thinking a foreign language as compared in their native language (Białek, 2023; McFarlane et al., 2020). However, these last authors did not provide specific hypotheses nor experimental evidence on how a foreign language could potentially alter metacognitive processes. Given that a crucial issue for dual-process theory concerns the circumstances under which analytic processes intervene to alter a heuristic output, it has been proposed that the process by which reasoners become aware of the need for such intervention is a metacognitive one (Thompson, 2009). Thus, these pieces of evidence strongly warrant a systematic empirical investigation of the metacognitive aspect of the FLE.

### 1.4 Metacognition in decision making

Metacognition, often defined as “thinking about thinking”, refers to the mental processes that monitor and control ongoing cognitive endeavors, apart from the gathered knowledge about the functioning of these cognitive endeavors (Dunlosky & Metcalfe, 2009; Flavell, 1979). The processes operate at the meta-level, overseeing cognitive operations at the object-level to evaluate their functionality (i.e., metacognitive monitoring) and to display the necessary resources or strategies (i.e., metacognitive control) (Dunlosky & Metcalfe, 2009; Nelson, 1990), depending on the monitoring and the goals. Metacognition plays a significant role in guiding human reasoning and decision-making, as its monitoring processes assess the unfolding and likelihood of success of cognitive processes, before it controls the initiation or halt of mental exertions based on the states of certainty or uncertainty regarding the cognitive activity in course. For this reason, enhanced metacognitive abilities such as monitoring and control are often linked to academic and professional success (Flavell, 1979; Vrugt & Oort, 2008). Therefore, it is crucial to understand how contextual factors, such as language, can influence processes at the metacognitive level that eventually lead to consequences at the cognitive level.

Meta-reasoning is a sub-field of metacognition applied to decision-making and problem solving (Ackerman & Thompson, 2017). One critical aspect of meta-reasoning is the monitoring of the reasoning process, which involves making judgments and assessments about the probability of success or failure in a given task. These monitoring processes, which are mostly spontaneous and implicit, provide looped feedback to the individual, allowing them to take appropriate control actions (Qiu et al., 2018; Schwarz, 2015). For example, if a person feels unsure about the rightness in their decision, they may choose to gather more information, take more time, or even change their approach to the problem. According to the theory of meta-reasoning, our answers to problems are accompanied by metacognitive experiences or judgments (Ackerman & Thompson, 2017; Cox & Raja, 2011). Such experiences can be assessed by recording individuals’ judgment of confidence, operationalized as the level of certainty about the accuracy of one’s own decision or performance (Fleming, 2024; Fleming & Lau, 2014; Rouault et al., 2018). The metacognitive judgment of certainty on initial intuitive answers in reasoning is conceptualized as the feeling of rightness (FOR), and the metacognitive judgment of certainty of the final answer is defined as the final judgment of confidence (FJC) (Ackerman & Thompson, 2017; Thompson et al., 2011). FOR is assumed to be an automatic experience-based response similar to affective responses, and therefore not subject to voluntary control (Koriat & Levy-Sadot, 1999). FOR and FJC represent distinct stages of metacognitive monitoring during the decision-making process, differing primarily in timing and function. FOR is an intuitive, immediate assessment occurring during or immediately after the decision-making process. It is based on heuristic cues and influences whether individuals will stick with or reconsider their initial response (Thompson, 2009). This heuristic-based assessment is rapid and often operates without extensive conscious deliberation (Ackerman & Thompson, 2017) and it is mostly based on fluency or ease of retrieval of the process itself and not the content of the response (Clavien & FitzGerald, 2017; Thompson, 2009). FJC is a reflective evaluation that occurs after the final decision has been made. FJC involves a deliberate assessment of the correctness of the final response made in the past, or sometimes referred to as error monitoring (Yeung & Summerfield, 2012). FJC, withing the meta-reasoning field, is considered essential for learning and self-regulation, helping individuals understand their cognitive strengths and weaknesses, therefore informing better strategies for future decisions (Ackerman & Thompson, 2017). In other words, FOR is an intermediate metacognitive judgment made before the definitive final decision that measures the degree to which the first solution that comes to mind feels right, whereas FJC is a metacognitive judgment made after the conclusion of the definitive decision that measures the subjective probability that the decision is correctly made. Generally, in cases where there are no decision reversals, meaning that the response is either generated in System 1 without System 2 engagement or that System 2 is engaged to rationalize the heuristic initial output, FJC should vary positively with FOR, reflecting no decision change. Whereas, in cases where there is a decision reversal, meaning that System 2 is engaged for redecision, one would expect relatively little or even negative relationship between FOR and FJC.

These meta-reasoning judgments serve as the basis in allocating and regulating mental resources in reasoning and decision making tasks at hand (Thompson, 2009; Thompson et al., 2011). Particularly relevant to our study is FOR, as the strength of FOR predicts the extent of System 2 reasoning (Simmons & Nelson, 2006; Thompson, 2009; Thompson et al., 2011; Vega et al., 2021). It is well established in moral psychology that System 2 is engaged in utilitarian reasoning, whereas System 1 is engaged in deontological reasoning (Greene et al., 2001; Greene et al., 2004). FOR thus serves as a trigger for engaging System 2. Research has demonstrated through various reasoning experiments that FOR judgments were consistently influenced by the fluency with which the initial answer was produced, providing a link to the Dual Process theory on the engagement of analytic thinking (Thompson et al., 2011, 2013; Wang & Thompson, 2019). For parallel comparison, the FOR in meta-reasoning is analogical to other metacognitive judgments such as the judgment of learning in metamemory, which are causally relevant in the decision between staying with the initial intuition or seek a more elaborated alternative (Mazzoni & Cornoldi, 1993; Nelson, 1993; Son, 2004; Son & Metcalfe, 2000). For example, Thompson (2009) proposed that initial intuitive answers are accompanied by a continuum of FORs, such that fluently generated items produce stronger FORs than their less fluent counterparts. In turn, it is the strength of FORs that determines the extent of subsequent analysis. In other words, the more compelling the initial answer, the lower the probability of subsequent analysis. Thompson et al. (2011) tested this hypothesis by asking reasoners to generate two responses to a series of reasoning problems: First, they were told to answer intuitively with the first answer that comes to mind and rate successively their FORs for these initial answers; then, they were allowed all the time required to give their final answers and rate successively their FJCs. The showed that the variability in the speed with which the initial answer was produced, an indicator of fluency, predicted the strength of FORs, such that fluently generated responses yielded strong FORs. During the rethinking period, people changed their answers between 10 to 30% of the time, depending on the task. However, regardless of the rate of change, the probability that the answer changed was determined by FORs, such that stronger FORs were accompanied by lower probabilities of change and vice versa. Similarly, the amount of time spent reconsidering the initial answer varied as a function of FORs, such that longer rethinking periods were preceded by weaker FORs and shorter rethinking periods by strong FORs. In conclusion, FORs predict both the extent and outcome of analytic engagement, aligning with the notion that metacognitive experiences significantly influence cognitive processes in decision-making.

One of the most well-established factors that influence metacognitive processes is cognitive load, because both cognitive and metacognitive processes draw from the same pool of mental resources (Norman & Bobrow, 1975). In other words, as the amount of mental resources for processes at the cognitive level increases, the available resources for processes at the metacognitive level decreases, thereby compromising the decision-making process. FOR, like other memory-based metacognitive judgments, is influenced by cue characteristics such as fluency that impact the retrieval experience (Koriat et al., 2004). In the realm of moral decisions, it is posited that the most prominent determinant of the strength of FORs is fluency: When a moral decision is fluently generated, the individual will have a strong and confident response; whereas, if it is generated less fluently, the individual will have a weak, unconfident response (Cecchini, 2023). This notion corroborates the broader literature on how processing fluency can impact a variety of judgments (Schwarz, 2004). For example, making a stimulus difficult to perceive can affect judgments of aesthetic pleasure (Reber et al., 2004), judgments of truth (Reber & Schwarz, 1999), and analytic reasoning (Alter et al., 2007). Similarly, extensive metamemory research has demonstrated that the ease of memory retrieval significantly influences the perceived accuracy of the memory (Benjamin et al., 1998; Matvey et al., 2001; Whittlesea & Leboe, 2003). Furthermore, studies have shown that the speed of answer generation strongly correlates with the confidence in memory retrieval accuracy (Kelley & Lindsay, 1993; Robinson et al., 1997). It is important to note that metacognitive judgments do not depend on the nature of the response itself but are second-order judgments about the retrieval experience. For instance, Vega et al. (2021) found that the type of moral judgment (deontological vs. utilitarian) did not consistently predict meta-reasoning experiences such as FOR. However, the level of FOR can impact subsequent System 2 engagement in reanalyzing the initial response, potentially leading to decision reversals. In fluent conditions, both deontological and utilitarian responses can be accompanied by high FOR, resulting in little System 2 engagement and few decision reversals. In disfluent conditions, lower FOR prompts more System 2 engagement, swaying decisions towards utilitarian responses. In sum, metacognition is crucially important in reasoning and decision making, and as it can be affected by cognitive load, contextual changes in this aspect can negatively impact the normal functioning of the monitoring and control processes.

### 1.5 Does a foreign language impact metacognitive processes such as meta-reasoning?

Despite the burgeoning literature on the FLE, previous research has not specifically addressed the issue of whether or not a foreign language impacts metacognition, though metacognition is crucial in human decision-making. Several studies in FLE research have incorporated metacognitive measures, but these studies have not focused on the metacognitive aspect of these differences between native and foreign languages in relation to the behavioral results. For example, Geipel et al. (2015a) asked participants to judge the wrongness of several moral dilemmas, e.g., consensual incest, in either the native or the foreign language and state their confidence in their judgments. Other than observing that participants judged these moral wrongdoings as less severe in the foreign language, they also reported that confidence ratings were much lower in the foreign language compared to the native language. Although these authors did not directly address the issue of whether a foreign language impacts metacognition, they did point out the possibility that a foreign language can increase uncertainty, thus prompting people to think more deliberately. Indeed, it was shown that deliberate thinkers show lower levels of overconfidence compared to intuitive thinkers (Mata et al., 2013). Furthermore, in a misinformation paradigm Dolgoarshinnaia and Martín-Luengo (2021) asked participants to attribute the information to their original source in native and foreign languages. These authors found that confidence ratings associated with incorrect attributions were much higher in the foreign language than in the native language, meaning that people seemed to be overconfident in their mistakes in a foreign language. The results of these two studies, Geipel et al. (2015a) and Dolgoarshinnaia and Martín-Luengo (2021)’s, appear to be in discord with each other concerning the metacognitive measure of confidence. More specifically, the former proposed that a foreign language could induce lower confidence, thus leading to better monitoring of their cognitive processes, whereas the latter suggested that a foreign language might lead to overconfidence in some aspect of their cognitive processes such as memory, therefore leading to worse monitoring of their answers. Contributing to the ambiguity surrounding this debate, one study reported no significant differences in confidence ratings for the cognitive reflection test in native and foreign languages, but they did observe a difference in the willingness to elaborate more as a function of foreign language proficiency (Mækelæ & Pfuhl, 2019), suggesting that while a foreign language may not impact monitoring, it can wreak consequences in control. Critically, these studies have only measured the judgment of confidence after the final decision, which is equivalent to FJC in the meta-reasoning literature, while ignoring FOR. Thus, it is difficult to draw conclusions on the impact of a foreign language on meta-reasoning, as FOR and FJC measure the different stages of monitoring in meta-reasoning.

To sum up, there is not yet consensus on how and in which direction a foreign language influences meta-reasoning. However, these pieces of evidence indisputably indicate that metacognitive experiences are somewhat different in a foreign language compared to a native language, echoing the propositions by some authors that distortion in metacognition could be an important driving factor of the FLE (Białek, 2023; McFarlane et al., 2020). In addition, existing theories like the reduced emotion hypothesis and the increased deliberation hypothesis converge on the same outcome, suggesting a common mechanism underlying these hypotheses. More specifically, decreased emotional resonance, increased disfluency, and increased System 2 engagement are all independent events, we propose that alterations in meta-reasoning in the foreign language can explain how and when this engagement occurs. Therefore, it is important to investigate the metacognitive aspect of the FLE, meta-reasoning, for we can gain further insight into how language could potentially enhance or impair human reasoning and decision making.

### 1.6 Study overview

In this research, our main objectives are to elucidate whether and how thinking in a foreign language affects metacognition, particularly in the monitoring and control processes of meta-reasoning. To attain this, we employ the moral decision-making paradigm, as a significant portion of FLE research pertains to moral dilemmas (Białek et al., 2019; Brouwer, 2019; Chan et al., 2016; Corey et al., 2017; Costa et al., 2014b; Geipel et al., 2015b, 2015a; Hayakawa et al., 2017; Miozzo et al., 2020). Two studies were conducted testing two different sets of dilemmas to better capture the variability in metacognitive processes in moral dilemma resolution in native and foreign languages.

In Study 1, we used a set of 24 moral dilemmas with non-repeating themes from the study by Greene et al. (2008). This set contains the 12 high-conflict personal dilemmas, e.g., the Footbridge dilemma (Foot, 1967), and 12 impersonal dilemmas, e.g., the Trolley dilemma (Thomson, 1985). The critical dilemmas for investigating the tension between utilitarian and deontological (or non-utilitarian) tendencies are high-conflict personal moral dilemmas, in which these two philosophical perspectives enter in conflict at a neurological level (Greene et al., 2004; Koenigs et al., 2007). In these high-conflict personal dilemmas, the sacrifice involved a “personal force” of the actor (e.g., “smothering a child”, “beheading someone”, etc., see the Supplementary Materials), as used in previous studies of FLE on moral dilemmas (e.g., Geipel et al., 2015a). Since these high-conflict dilemmas share a similar structure in which one person can be harmed in order to achieve a greater benefit, to avoid repetition and to offer comparison, we presented impersonal moral dilemmas as filler scenarios and a control condition (Greene et al., 2001, 2004). Although FLE has been typically observed in personal dilemmas rather than impersonal dilemmas (Cipolletti et al., 2016; Corey et al., 2017; Stankovic et al., 2022), we included impersonal dilemmas in the design to broaden the spectrum of scenarios as well as to have a baseline or control measure. This set of moral dilemmas not only introduces a broad spectrum of scenarios to better capture the variability in metacognitive processes but also significantly expands the stimuli set traditionally used in the metacognitive and FLE studies of moral decisions.

In Study 2, the set consists of a battery of dilemmas that allows the adoption of the Process Dissociation approach to account for relative weighting of the deontological and utilitarian tendencies, providing a more nuanced measure of the moral foreign language effect. This approach involves presenting each participant with incongruent and congruent moral dilemmas. Incongruent dilemmas, such as deciding to sacrifice one person to save five, have differential outcomes: deontology forbids sacrificing one person to save others, while utilitarianism supports it. In contrast, congruent dilemmas are structured similarly but feature outcome alignment between deontological and utilitarian perspectives. For example, if the choice involves sacrificing one person to prevent five others from experiencing minor injuries, both ethical frameworks would oppose the sacrifice. Study 2^1^ aims also to replicate and expand the results of Study 1.

We made several predictions in this registered report. First, for Study 1, aligning with previous research in FLE, we predicted that people would be more likely to make utilitarian decisions for personal moral dilemmas (e.g., Footbridge dilemma) in a foreign language compared to their native language; for Study 2, we predicted that the foreign language will decrease deontological responses but not increase utilitarian responses. Second, for both studies, since processing a foreign language is generally less fluent in processing one’s native language (Abutalebi, 2008; Hadjichristidis et al., 2017; Volk et al., 2014), we predicted that people would take much longer time to generate their decisions in the foreign language than their native language. Third, because reduced fluency leads to decreased FORs (Cecchini, 2023; Thompson, 2009), we predicted that people would have lower FORs in a foreign language compared to their native language. Fourth, since decreased FORs are associated with increased successive reanalysis (Thompson et al., 2011; Vega et al., 2021), we also predicted that FORs would be negatively correlated with redecision time, i.e., lower FORs would be associated with longer decision times and vice versa. Fifth, similarly we predicted that FORs would be negatively correlated with decision reversals, i.e., lower FORs would be associated with more decision reversals and vice versa. Additionally, given that individuals with higher proficiency in the foreign language are more fluent and thus less affected by the FLE (Costa et al., 2014b; Del Maschio et al., 2022; Stankovic et al., 2022), we predicted that foreign language proficiency would modulate these effects, such that people with higher foreign language proficiency would exhibit less impact of FLE in decisions, decision times, and FORs. We did not hold specific predictions regarding FJCs, but they were measured for the completeness of the study.

To evaluate these hypotheses, the present study adopted Thompson et al.’s (2011) experimental procedure to investigate the metacognitive processes during moral decision making. This paradigm was employed to delve into the metacognitive dynamics of moral judgment, measuring initial/fast and final/slow responses, with decision times and redecision times serving as indicators of controlled System 2 engagement.

## 2. Study 1

### 2.1 Methods

In this study, we aimed to investigate the effect of Language on several decision-making parameters. We manipulated Language (native vs. foreign) as a between-subjects factor, meaning that half of the participants completed the task in their native language (i.e., English), and the other half in a foreign language (i.e., Spanish). We sought to examine the effect of Language on Decisions (utilitarian vs. deontological), Decision Times, Feeling of Rightness (FOR), Redecision Time, and Final Judgment of Confidence (FJC). The experiment was approved by the Ethics Committee of the University of Padua (Protocol: 289-b) and was carried out in accordance with The Code of Ethics of the World Medical Association (Declaration of Helsinki) for experiments involving humans.

### 2.2 Materials

We used a set of 24 moral dilemmas with non-repeating themes from the study by Greene et al. (2008). This set contains the 12 high-conflict personal dilemmas, e.g., the Footbridge dilemma (Foot, 1967), and 12 impersonal dilemmas, e.g., the Trolley dilemma (Thomson, 1985). Since the original dilemmas from Greene et al. (2008) are in English, a Spanish version of these dilemmas was created by one of the researchers and proof-read by two other native speakers of Spanish (see the Supplementary Materials).

### 2.3 Participants

In our registered report, we employed a power simulation to accurately determine the requisite sample size for our study using the package simr (Green & MacLeod, 2016). We coded the conditions as follows: ‘0’ for the native language and ‘1’ for the foreign language. The model utilized for this power simulation is a linear mixed effects model, incorporating Language as a fixed effect, proficiency and Participant and Trial as random effects. Since there was no previous evidence regarding inter-item and inter-participant consistency with such a design, we adopted a moderate stance by setting the Intraclass Correlation Coefficients for both participants and dilemmas at a medium value of 0.5.

Two power simulations were performed on the desired sample size according to the dependent variable, with an alpha level of 0.05 and a target power of 0.8. For the effect of Language on Decision (formula: Decision ~ Language + (1 | Participant) + (1 | Trial), family = binomial(link = “logit”)), the sample size was calculated with an effect size of 1.64 OR (Stankovic et al., 2022), indicating a sample size of 125 participants. For the effect of Language on FOR (formula: FOR ~ Language + (1 | Participant) + (1 | Trial), family = gaussian(link = “identity”)), the sample size was calculated with a medium effect size (Cohen’s d = 0.4), though previous research reported a large effect (Cohen’s f = 0.38) for the effect of Language on confidence, indicating 115 participants. All simulated data and the power analysis is available in the OSF repository (osf.io/m7kc8).

Taking possible exclusion of participants into account, 140 participants would be recruited via Prolific. We followed similar screening and exclusion criteria that have been used in past work on foreign language effects (Costa et al., 2014b; Keysar et al., 2012). Only participants that passed the following pre-screeners were eligible to participate in this study: (a) first language being English, (b) earliest language in life being English, (c) being raised with English only, (d) primary language being English, (e) currently residing in the US, and (f) fluent in Spanish. Therefore, the target population were sequential bilinguals and not simultaneous bilinguals, aligning with the demographic profile of previous FLE research.

### 2.4 Procedure

This procedure closely adhered to the methodology outlined by Thompson et al. (2011) and successively by Vega et al. (2021). Participants were required to complete the experiment on a computer screen in a quiet environment. After consenting to participation in the experiment, participants were randomly assigned to complete the experiment either in their native language or the foreign language.

Eligible participants went through a second phase of screening mirroring the procedure of Hayakawa et al. (2017). They were randomly given one of two comprehension quizzes in the assigned language prior to completing the experiment and the other quiz in the other language after completing the experiment. The comprehension quizzes involved reading a paragraph and answering a multiple-choice question about what they had just read. Only participants who answered the first question correctly were allowed to continue the experiment.

**Figure 1:**
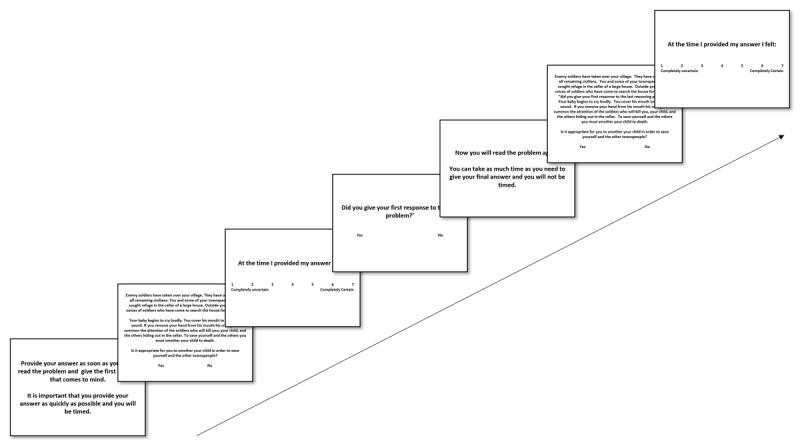
Experimental procedure for a single trial for the Decision-Redecision Paradigm.

They read the instructions before commencing the experimental trials. In each trial, participants were instructed to provide their immediate and initial response to the dilemma. Then, participants read a moral dilemma, and made a forced choice decision between “Yes” and “No” regarding the appropriateness of the utilitarian action as a resolution for the dilemma by clicking the corresponding button below each dilemma. “Yes” denotes a utilitarian response and “No” denotes a deontological response. Subsequent to their initial response, participants indicated a FOR by responding to the prompt, “At the time I provided my answer I felt:” with a Likert scale ranging from 1 (completely uncertain) to 7 (completely certain) (see Thompson et al., 2011). A manipulation check ensued, inquiring if participants had indeed offered their immediate response. Successively, participants were instructed to take all the time they require to give their final decision on the dilemma. Then, they read the dilemma again with the same decision task. And once again, after the decision was made, they indicated a FJC using the aforementioned scale. Each of the 24 experimental trials consisted of a dilemma presented in the middle of the screen (Lato font, size 16, and black color in bold) on white background (see Figure 1). After the main task, participants completed a questionnaire regarding their demographic information, including age and gender, and regarding their language background, including native language proficiency (7-point Likert scale), foreign language proficiency (7-point Likert scale), age of acquisition, and exposure (7-point Likert scale). The questionnaire was in English for all the participants. The experiment was implemented on Labvanced (https://www.labvanced.com). All stimuli and the questionnaire are openly available in the OSF repository (osf.io/m7kc8).

### 2.5 Data Analysis and strategies

Main analyses were performed using the lme4 package (Bates et al., 2015) in R software (R Core Team, 2022). Only the data of participants that answered correctly to both the pre- and post-experiment comprehension quizzes were considered for analysis. We first performed an outlier detection in total experiment completion times: participant’s mean by more or less than 2.5 standard deviations were excluded from successive analysis. Further, the trials where participants indicated that they had not provided the first answer that came to mind were excluded from further analysis. Decision reversals in the critical trials was counted for each participant. A “relative proficiency” (RP) score was calculated by subtracting the foreign language proficiency rating from the native language proficiency rating (Hayakawa et al., 2017). A high score thus indicates that the foreign language proficiency is notably lower than that of the native tongue.

Mixed effects linear models were performed for the effects of Language (native vs. foreign), and Dilemma (personal vs. impersonal) on Decision, Decision time (DT), Redecision time (RDT), FORs, FJCs, and Decision reversals (DR), as well as the modulating effect of proficiency (RP) on the dependent variables in the foreign language condition. By “Decision” we refer to the participant’s selection between options in a moral dilemma scenario (“yes” vs. “no”), coded as a binary outcome. The effect of Language and Dilemma on Decision was analyzed using this formula: Decision ~ Language * Dilemma + (1 | Participant), family = binomial(link = “logit”). The effect of proficiency (RP) on Decision in the foreign language condition was analyzed using this formula: Decision ~ RP + (1 | Participant), family = binomial(link = “logit”).

The effect of Language and Dilemma on Decision time (DT) and Redecision Times (RDT) was measured using this formula: DT/RDT ~ Language * Dilemma + (1 | Participant), family = inverse.gaussian(link = “identity”). The effect of proficiency (RP) on DTs and RDTs in the foreign language condition was analyzed using this formula: DT/RDT ~ RP + (1 | Participant), family = inverse.gaussian(link = “identity”).

The effect of Language and Dilemma on FORs and FJCs was analyzed using this formula: FOR/FJC ~ Language * Dilemma + (1 | Participant), family = gaussian(link = “identity”). The effect of proficiency (RP) on FORs and FJCs in the foreign language condition was analyzed using this formula: FOR/FJC ~ RP + (1 | Participant), family = gaussian(link = “identity”).

The effect of Language and Dilemma on Decision reversals (DR) was analyzed using this formula: DR ~ Language * Dilemma + (1 | Participant), family = poisson(link = “log”). The effect of proficiency (RP) on DR in the foreign language condition was analyzed using this formula: DR ~ RP + (1 | Participant), family = poisson(link = “log”).

In addition to the mixed models for the main analyses, we also analyzed correlations to assess the relationships between FORs and Decision time (DT), Redecision time (RDT), or Decision reversals (DR) to ease the comparison with previous research (Thompson et al., 2011). To this end, correlations were performed using this formula: cor(DT/RDT/DR, FORs, method = “spearman”).

## 3. Study 2

### 3.1 Methods, participants, and procedure

The methods, participants sample, and procedure mirrored that of Study 1.

### 3.2 Materials

The materials for Study 2 consists of a battery of impersonal dilemmas taken from Hayakawa et al. (2017), which were originally used in the Conway and Gawronski (2013) study. This set of moral dilemmas is composed by 10 congruent and 10 incongruent moral dilemmas. Since the original dilemmas from Hayakawa et al. (2017) are in English, a Spanish version of these dilemmas was created by one of the researchers and proof-read by two other native speakers of Spanish (see the Supplementary Materials).

### 3.3 Data analysis and strategies

The same outlier detection and “relative proficiency” (RP) score were calculated as in Study 1. In addition, we calculated a utilitarianism parameter (*U*) for each participant by taking the difference in the proportion of “no” responses between congruent trials and incongruent trials: *U* = *p* ( no ∣ congruent ) – *p* (no ∣ incongruent ). And we isolated the deontology parameter (*D*) for each participant by calculating the proportion of “no” responses in incongruent trials relative to all deontological responses: *D* = *p* ( no | incongruent ) / (1 – *U*).

Then, mixed effects linear models were performed for the effects of Language on *U* score, *D* score, Decision time (DT), Redecision time (RDT), FORs, FJCs, and Decision reversals (DR), as well as the modulating effect of proficiency (RP) on the dependent variables in the foreign language condition. The effect of Language on *U* score and *D* score was analyzed using this formula: D/U ~ Language + (1 | Participant), family = gaussian(link = “identity”). The effect of proficiency (RP) on *U* and *D* scores in the foreign language condition was analyzed using this formula: D/U ~ RP + (1 | Participant), family = gaussian(link = “identity”).^2^

The effect of Language on Decision Times (DT) and Redecision Times (RDT) was analyzed using this formula: DT/RDT ~ Language + (1 | Participant) + (1 | Trial), family = inverse.gaussian(link = “identity”). The effect of proficiency (RP) on DTs and RDTs in the foreign language condition was analyzed using this formula: DT/RDT ~ RP + (1 | Participant) + (1 | Trial), family = inverse.gaussian(link = “identity”).

The effect of Language on FORs and FJCs was analyzed using this formula: FOR/FJC ~ Language + (1 | Participant) + (1 | Trial), family = gaussian(link = “identity”). The effect of proficiency (RP) on FORs and FJCs in the foreign language condition was analyzed using this formula: FOR/FJC ~ RP + (1 | Participant) + (1 | Trial), family = gaussian(link = “identity”).

The effect of Language on Decision reversals (DR) was analyzed using this formula: DR ~ Language + (1 | Participant) + (1 | Trial), family = poisson(link = “log”). The effect of proficiency (RP) on DR in the foreign language condition was analyzed using this formula: DR ~ RP + (1 | Participant) + (1 | Trial), family = poisson(link = “log”).

The correlations between FORs and Decision time, Redecision time, or Decision reversals will be analyzed using this formula: cor(DT/RDT/DR, FOR, method = “spearman”).

## 4. Results and discussion of Study 1

A total of 157 Prolific workers completed this experiment out of 214 that participated. Five participants were excluded from further analysis for failing the comprehension checks and two additional participants were excluded for being outliers in terms of reaction times, resulting in 150 eligible participants (72 females, 77 females, and 1 non-binary person). Ninety-one participants completed the experiment in the native language condition and 59 completed it in the foreign language condition.

A Welch Two Sample t-test was conducted to compare the mean ages between the native and foreign language groups. The results indicated no statistically significant difference in mean age between the two groups in Age (*p* = .753), Age of Acquisition of FL (*p* = .308), NL proficiency (*p* = .769), FL proficiency (*p* = .063), and FL exposure (*p* = .520). See Table 1 for demographic and linguistic background of the participant pool.

**Table 1:** Demographic and linguistic background of the participant pool in Study 1.


	MEAN	SD

Age	32.83	9.10

Age of Acquisition of FL	6.77	6.31

NL proficiency (7 = highest)	6.99	0.12

FL proficiency (7 = highest)	5.53	1.13

FL exposure (7 = highest)	5.35	1.70


### 4.1 Decision outcomes

**Figure 2:**
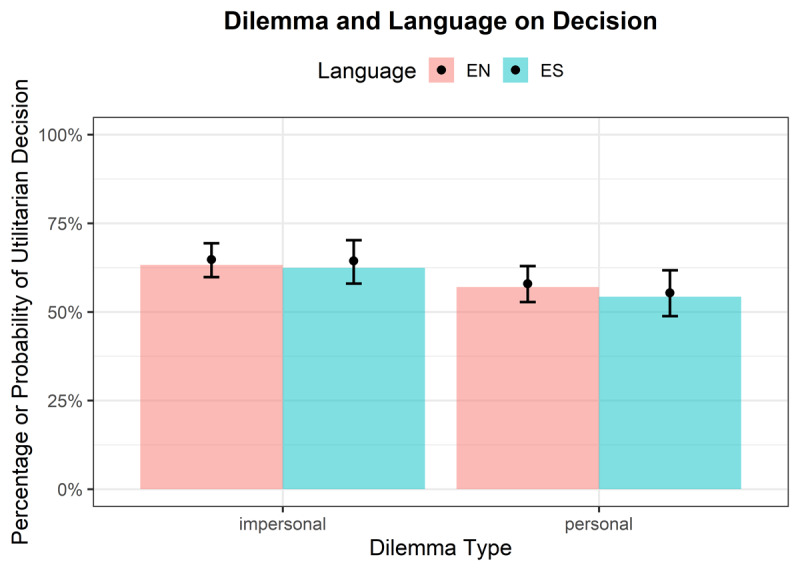
Interaction of Language and Dilemma on Decision with bars showing raw proportions and lines showing model-predicted probabilities with 95% CIs.

**Decision**. A statistically significant main effect was observed for Dilemma type (OR = 0.75, 95% CI [0.63, 0.90], *p* = .002), indicating that the odds of making a utilitarian decision were 24% lower for personal dilemmas compared to impersonal dilemmas (see Figure 2). Neither the main effect of Language (OR = 0.98, 95% CI [0.70, 1.38], *p* = .922) nor the interaction between Language and Dilemma (OR = 0.92, 95% CI [0.68, 1.23], *p* = .559) was statistically significant. The Intraclass Correlation Coefficient (ICC) was 0.16, suggesting that 16% of the variance in initial moral decisions on the log-odds scale was attributable to individual differences among subjects. The effect of proficiency was not significant (OR = 1.07, 95% CI [0.84, 1.36], *p* = .922).

**Figure 3:**
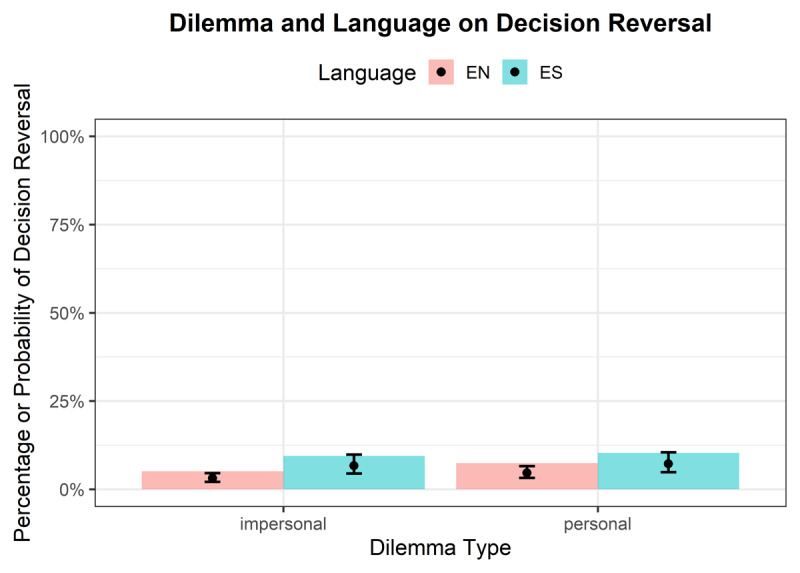
Interaction of Language and Dilemma on Decision Reversal with bars showing raw proportions and lines showing model-predicted probabilities with 95% CIs.

**Decision Reversals**.^3^ A statistically significant main effect was observed for Dilemma type (OR = 1.52, 95% CI [1.05, 2.19], *p* = .026), indicating that the odds of reversing a decision were higher for personal dilemmas compared to impersonal dilemmas. A statistically significant main effect was also observed for Language (OR = 2.24, 95% CI [1.28, 3.91], *p* = .005), indicating that the odds of reversing a decision were higher in the foreign language (see Figure 3). However, the interaction between Language and Dilemma (OR = 0.71, 95% CI [0.42, 1.21], *p* = .209) was not statistically significant. The ICC was 0.27. The effect of proficiency was not significant (OR = 1.08, 95% CI [0.76, 1.52], *p* = .680).

### 4.2 Decision Times

**Figure 4:**
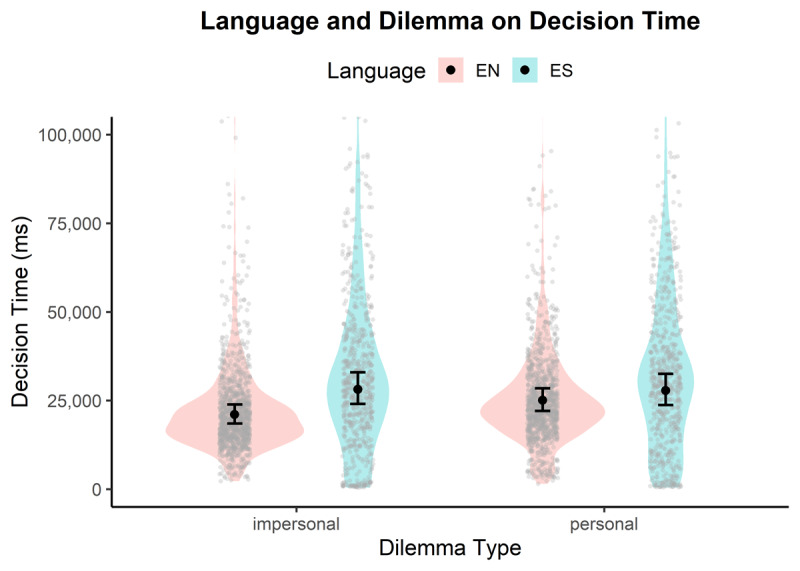
Interaction of Language and Dilemma on Decision Time with violins showing raw data distribution and lines showing model-predicted means with 95% CIs.

**Decision time**. A statistically significant main effect was observed for Dilemma (*β* = 1.19, 95% CI [1.14, 1.25], *p* < .001), indicating that decision times were much longer for personal dilemmas compared to impersonal dilemmas. A statistically significant main effect was also observed for Language (*β* = 1.34, 95% CI [1.09, 1.64], *p* = .005), indicating that decision times were much longer in the foreign language condition compared to the native language (see Figure 4). Furthermore, the interaction between Language and Dilemma was statistically significant (*β* = 0.83, 95% CI [0.77, 0.89], *p* < .001), suggesting that the increase in reaction time attributed to the foreign language was attenuated in personal dilemmas, compared to their purely additive effects (see Figure 4). The effect of proficiency was not significant (*β* = 1.10, 95% CI [0.92, 1.32], *p* = .280).

**Redecision time**. A statistically significant main effect was observed for Dilemma (*β* = 1.15, 95% CI [1.06, 1.25], *p* = .001), indicating that redecision times were much longer for personal dilemmas (*M* = 12188 ms, *SD* = 21540 ms) compared to impersonal dilemmas (*M* = 11573 ms, *SD* = 24578 ms). A statistically significant main effect was also observed for Language (*β* = 2.21, 95% CI [1.61, 3.03], *p* < .001), indicating that redecision times were much longer in the foreign language condition (*M* = 16584 ms, *SD* = 31512 ms) compared to the native language (*M* = 8989 ms, *SD* = 15186 ms). Furthermore, the interaction between Language and Dilemma was statistically significant (*β* = 0.81, 95% CI [0.70, 0.93], *p* = .002), suggesting that the increase in reaction time attributed to the foreign language was attenuated in personal dilemmas, compared to their purely additive effects. The effect of proficiency was not significant (*β* = 1.04, 95% CI [0.84, 1.27], *p* = .735).

### 4.3 Metacognition

**Figure 5:**
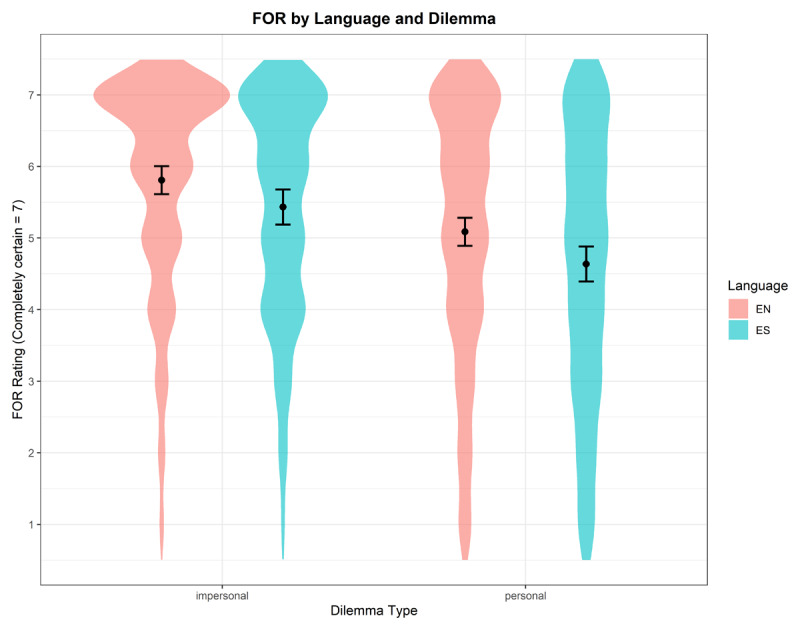
Interaction of Language and Dilemma on FOR with violins showing raw data distribution and lines showing model-predicted means with 95% CIs.

**FOR**. A statistically significant main effect was observed for Language (*β* = –0.37, 95% CI [–0.69, –0.06], *p* = .019), with this negative coefficient indicating that FOR was lower in the foreign language condition compared to in the native language (see Figure 5). A statistically significant main effect was also observed for Dilemma (*β* = –0.72, 95% CI [–0.84, –0.60], *p* < 0.001), where the negative coefficient suggests that the outcome variable was lower for personal dilemmas compared to for impersonal dilemmas. The interaction between Language and Dilemma was not statistically significant (*β* = –0.08, 95% CI [–0.27, 0.12], *p* = 0.449). The ICC was 0.26. The effect of proficiency was not significant (*β* = –0.19, 95% CI [–0.41, 0.04], *p* = .104).

**FJC**. A statistically significant main effect was observed for Language (*β* = –0.39, 95% CI [–0.70, –0.08], *p* = .014), with this negative coefficient indicating that FJCs were lower in the foreign language condition (*M* = 5.28, *SD* = 1.73) compared to in the native language (*M* = 5.66, *SD* = 1.60). A statistically significant main effect was also observed for Dilemma (*β* = –0.71, 95% CI [–0.83 – –0.60], *p* < .001), where the negative coefficient suggests that FJCs were lower for personal dilemmas (*M* = 5.15, *SD* = 1.78) compared to for impersonal dilemmas (*M* = 5.88, *SD* = 1.44). The interaction between Language and Dilemma was not statistically significant (*β* = –0.01, 95% CI [–0.20, 0.18], *p* = .926). The ICC was 0.28. The effect of proficiency was significant (*β* = –0.24, 95% CI [–0.48, –0.01], *p* = .038).

### 4.4 Correlations with FOR

**Decision time**. A GLM^4^ with a Gamma family and log link was fitted to test the relationship between Decision Times and FOR. Decision Time showed a statistically significant negative relationship with FOR (*β* = 0.94, 95% CI [0.93, 0.96], *p* < .001), indicating that higher Decision Times were associated with lower FOR scores.

**Figure 6:**
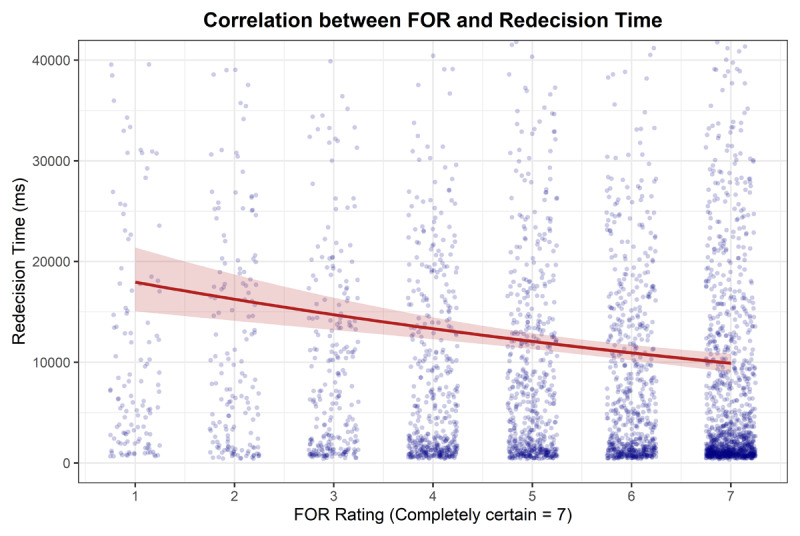
Correlation between FOR and Redecision Time showing Raw data with fitted line from Gamma GLM and 95% CIs.

**Redecision time**. A GLM with a Gamma family and log link was fitted to predict Redecision Time from FOR. FOR showed a statistically significant negative relationship with Redecision Time (*β* = 0.91, 95% CI [0.87, 0.94], *p* < .001). This indicates that as FOR increase Redecision Time decreases (see Figure 6).

**Decision reversal**. A GLM with a binomial family and logit link was fitted to predict DR from FOR. FOR showed a statistically significant negative relationship with DR (OR = 0.68, 95% CI [0.64, 0.73], *p* < .001), indicating that as FOR increases the probability Decision Reversal decreases.

### 4.5 Discussion

The primary objective of Study 1 was to investigate the FLE in metacognition (viz., meta-reasoning), positing that the cognitive disfluency experienced in processing stimuli in a foreign language would impact metacognitive monitoring and control in decision-making. We used moral decisions as these had been studied extensively leading to the emergence of the so-called “moral FLE” (mFLE). While our findings of Study 1 did not replicate the classic mFLE on decision outcomes, they provide compelling evidence for a FLE at the metacognitive level, offering strong support for our hypothesis.

Consistent with studies in moral psychology (e.g., Greene et al., 2001, 2004), our results confirmed a robust main effect of dilemma type. Participants were significantly more likely to endorse utilitarian actions and responded more quickly when faced with impersonal dilemmas compared to personal dilemmas. This validates our materials and confirms that personal dilemmas elicited greater cognitive and emotional conflict. However, contrary to some previous research (e.g., Corey et al., 2017; Costa et al., 2014b), where participants were supposedly more utilitarian in personal dilemmas (i.e., Footbridge) in a foreign language, we found no significant main effect of language, nor an interaction of Language and Dilemma on decision outcomes. The null results of this registered report challenges the robustness of the mFLE, as a growing number of studies reported either null effects (Białek et al., 2019; Brouwer, 2019; Čavar & Tytus, 2017; Dylman & Champoux-Larsson 2020; Flexas et al., 2023; Teitelbaum Dorman et al., 2024), mixed effects (Chan et al., 2016; Privitera, 2024; Xu et al., 2024), or even reversed effects (Fitzgerald et al., 2025; Mills & Nicoladis, 2020; Muda et al., 2018; Wong & Ng, 2018). In summary, these results conjointly allude to a publication bias in the mFLE research, strongly warranting targeted analyses including also unpublished results.

In stark contrast, the results for our processing and metacognitive measures aligned closely with our hypotheses. A significant main effect of language revealed that decision times were substantially longer in the foreign language condition, serving as a proxy to confirm elevated cognitive disfluency in a foreign language. Critically, this increased cognitive disfluency impacted metacognitive monitoring in predicting decreased FORs in the foreign language condition, indicating that participants felt more uncertain about their decisions when reading in their foreign language. Furthermore, the decreased FORs in the foreign language condition appeared to trigger more metacognitive control, as evidenced by significantly longer redecision times and a higher rate of Decision Reversals. Our results on FJCs corroborate previous findings on retrospective confidence (Dolgoarshinnaia and Martín-Luengo, 2021; Geipel et al., 2015a) and further support the notion that reasoning in a foreign language could lead to disruptions in metacognition. Finally, across both language contexts, we replicated the foundational findings of the meta-reasoning framework (Thompson et al., 2011; Vega et al., 2021): FOR was significantly and negatively correlated with decision time, redecision time, and the likelihood of decision reversals, confirming its role in triggering more System 2 engagement.

In addition to uncovering a FLE on metacognition, our results suggest that focusing solely on the final decision outcomes may obscure the complex ways in which a foreign language influences human mind. The robust effects on processing time, confidence, and decision reversals provide strong evidence for our metacognitive hypothesis in FLE. While the ultimate decision remained unchanged, the pathway to that decision was different. The lower FOR in the foreign language condition directly supports the thesis that reduced processing fluency acts as an informative metacognitive cue, signaling uncertainty and triggering a need for further cognition (Alter et al., 2007; Thompson, 2009). The consequent increase in redecision time and decision reversals demonstrates the behavioral consequence of this metacognitive signal: participants engaged in more System 2 processing to scrutinize and, in some cases, overturn their initial intuitions. This positions our work as a key contribution to the field, that is, the FLE may not be an “all-or-nothing” phenomenon that invariably flips a decision from deontological to utilitarian. Rather, its primary effect may lie in modulating the metacognitive system that governs the allocation of cognitive resources (cf. Mækelæ & Pfuhl, 2019). This perspective helps reconcile the often-mixed results in the FLE literature; the impact on final choice may depend on whether the increased deliberation, prompted by metacognitive uncertainty, is sufficient to overcome a prepotent intuitive response. Contrary to our predictions, relative proficiency (RP) did not emerge as a significant moderator in this study. This might be due to a relatively homogenous sample of speakers in Study 1, limiting our power to detect such an effect.

The present study has two principal limitations that Study 2 was designed to address. First, the use of a binary choice (utilitarian vs. deontological) is an inherently coarse measure that forces a trade-off between two competing concerns. This framework cannot distinguish whether the FLE works by weakening deontological inclinations, strengthening utilitarian reasoning, or both. Second, the classic dilemma set, while foundational, features highly evocative and perhaps familiar scenarios (e.g., the Footbridge dilemma), which could introduce unforeseen variance.

To overcome these limitations, Study 2 employs the Process Dissociation (PD) framework developed by Conway and Gawronski (2013). By using a different set of dilemmas composed of congruent and incongruent scenarios, this approach allows for the independent estimation of parameters for deontological and utilitarian inclinations. This provides a more nuanced lens through which to examine how language context affects the underlying components of moral reasoning. It is important to point out that there was no previous research combining the PDA and decision-redecision paradigm to test the metacognitive processes of moral decision-making, nevertheless, Study 2 aimed to offer a more nuanced understanding of the metacognitive account of the mFLE.

## 5. Results and discussion of Study 2

A total of 177 participants were recruited from Prolific to participate in experiment 2, out of which only 124 completed the experiment. One participant was excluded from further analysis for failing the comprehension checks and two additional participants were excluded for being outliers in terms of reaction times, resulting in 121 eligible participants (67 females, 49 males, and 5 non-binary individuals). Sixty-one participants completed the experiment in the native language condition and 60 completed it in the foreign language condition.

A Welch Two Sample t-test was conducted to compare the mean ages between the native and foreign language groups. The results indicated no statistically significant difference in mean age between the two groups in Age (*p* = .759), Age of Acquisition of FL (*p* = .391), NL proficiency (*p* = .984). However, FL proficiency is higher (*p* = .008) in the foreign condition (*M* = 5.68, *SD* = 0.95) than in the native condition (*M* = 5.15, *SD* = 1.22), and FL exposure (*p* = .026) is higher in the foreign condition (*M* = 5.97, *SD* = 1.25) than in the native condition (*M* = 5.38, *SD* = 1.61). See Table 2 for demographic and linguistic background of the participant pool.

**Table 2:** Demographic and linguistic background of the participant pool in Study 2.


	MEAN	SD

Age	30.72	8.74

Age of Acquisition of FL	6.64	6.26

NL proficiency (7 = highest)	6.95	0.22

FL proficiency (7 = highest)	5.41	1.12

FL exposure (7 = highest)	5.67	1.47


### 5.1 D-score and U-score

**D-score**. The effect of Language was not significant (*β* = –0.05, 95% CI [–0.12, 0.03], *p* = .233). The effect of proficiency was significant (*β* = –0.07, 95% CI [–0.13, –0.01], *p* = .033), indicating that participants less proficient in the foreign language showed less deontological tendencies.

**U-score**. The effect of Language was not significant (*β* = –0.03, 95% CI [–0.10, 0.04], *p* = .375). The effect of proficiency approached significance (*β* = –0.05, 95% CI [–0.10, 0.00], *p* = .067).

### 5.2 Decision times

**Decision time**. The effect of Language on Decision Time was significant (*β* = 1.61, 95% CI [1.53, 1.70], *p* < .001), indicating that participants spent much longer time making decisions in the foreign language (*M* = 31990 ms, *SD* = 22708 ms) compared to the native language (*M* = 19963 ms, *SD* = 14349 ms). The effect of proficiency on Decision Time approached significance (*β* = 0.79, 95% CI [0.63, 1.00], *p* = .052).

**Redecision time**. The effect of Language on Redecision Time was significant (*β* = 1.41, 95% CI [1.00, 1.99], *p* = .047), indicating that participants spent longer time in redecision in the foreign language (*M* = 12049 ms, *SD* = 17082 ms) compared to the native language (*M* = 8642 ms, *SD* = 15063 ms). The effect of proficiency on Decision Time was not significant (*β* = 0.93, 95% CI [0.71, 1.21], *p* = .576).

### 5.3 Metacognition

**Figure 7:**
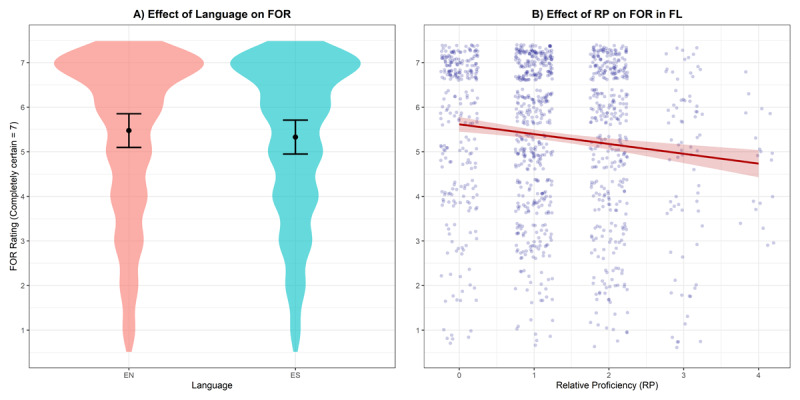
**(A)** The effect of Language on FOR with violins showing raw data distribution and lines showing model-predicted means with 95% Cis; **(B)** The effect of RP (relative proficiency) on FOR with jitters showing raw data points and model-predicted fitted line with 95% CIs.

**FOR**. The FOR values were slightly lower in the foreign language (*M* = 5.34, *SD* = 1.72) compared to the native language (*M* = 5.48, *SD* = 1.76), though this effect of Language was not significant (*β* = –0.15, 95% CI [–0.44, 0.15], *p* = .333). The Intraclass Correlation Coefficient (ICC) was 0.34. The effect of proficiency on FOR approached significance (*β* = –0.22, 95% CI [–0.45, 0.00], *p* = .054), suggesting a trend that as participants’ relative proficiency decreases their FORs decrease (see Figure 7).

**FJC**. The FJC values were slightly lower in the foreign language (*M* = 5.52, *SD* = 1.70) compared to the native language (*M* = 5.64, *SD* = 1.70), though the effect of Language on FJC was not significant (*β* = –0.13, 95% CI [–0.43, 0.16], *p* = .376). The ICC was 0.34. The effect of proficiency on FJC was significant (*β* = –0.27, 95% CI [–0.49, –0.05], *p* = .017), indicating that as participants’ relative proficiency decreases their FJCs decrease.

### 5.4 Decision Reversal

**Decision reversals**. The total percentage mean of decision reversals was 6.35% (*SD* = 2.24%). The effect of Language on DR was not significant (OR = 0.94, 95% CI [0.52, 1.72], *p* = .853). The ICC was 0.32. The effect of proficiency on DR was not significant (OR = 1.32, 95% CI [0.83, 2.08], *p* = .237).

### 5.5 Correlations with FOR

**Decision time**. A GLM with a Gamma family and log link was fitted to test the relationship between Decision Times and FOR. Decision Time showed a statistically significant negative relationship with FOR (*β* = 0.97, 95% CI [0.96, 0.99], *p* = .002), indicating that higher Decision Times were associated with lower FOR scores.

**Figure 8:**
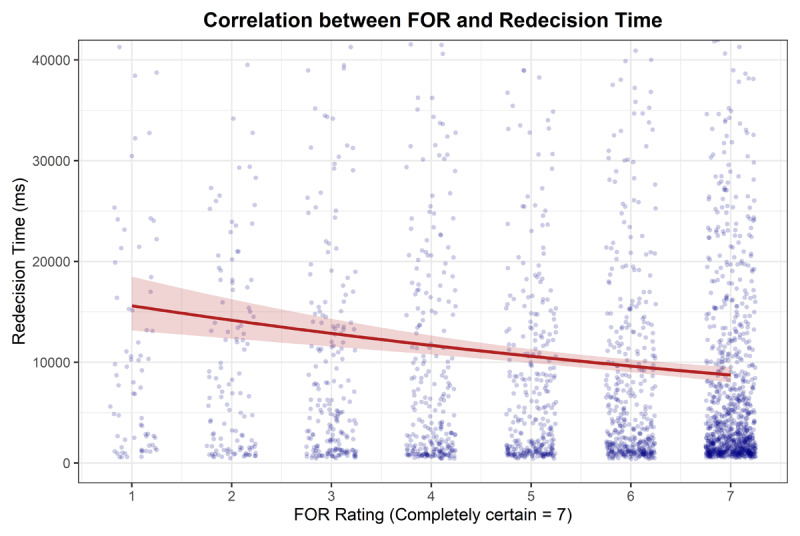
Correlation between FOR and Redecision Time showing raw data points with fitted line from Gamma GLM and 95% CIs.

**Redecision time**. A GLM with a Gamma family and log link was fitted to predict Redecision Time from FOR. FOR showed a statistically significant negative relationship with Redecision Time (*β* = 0.91, 95% CI [0.87, 0.94], *p* < .001). This indicates that as FOR increase Redecision Time decreases (see Figure 8).

**Decision reversal**. A GLM with a binomial family and logit link was fitted to predict DR from FOR. FOR showed a statistically significant negative relationship with DR (OR = 0.67, 95% CI [0.61, 0.72], *p* < .001), indicating that as FOR increases the probability Decision Reversal decreases.

### 5.6 Discussion

Building upon the findings of Study 1, the second experiment sought to dissect mFLE with greater precision by combining employing the Process Dissociation (PD) framework. This approach allowed for the independent measurement of utilitarian and deontological inclinations, aiming to clarify whether experimental language affects deontology and utilitarianism differentially.

Contrary to previous studies reporting decreased deontology in a foreign language (Hayakawa et al., 2017; Peressotti et al., 2024), we observed no such effect. Nonetheless, Relative Proficiency (RP)—the gap between native and foreign language proficiency—was a significant negative predictor of the D-score, indicating that individuals less proficient in the foreign language (vs. their native language) relied less on their deontological inclinations. This partially corroborates previous research showing that FL reduces emotional resonance (Geipel et al., 2015b) and norm adherence (Hu & Navarrete, 2024), factors associated with deontology. However, this interpretation should be considered cautiously, as other research also suggested that a foreign language could increase utilitariansim (Kirova et al., 2023) or even reduce both deontological and utilitarian tendencies (Muda et la., 2018). The null results of FLE on moral inclinations per se is noteworthy. Referring back to our discussion for Study 1, these findings suggest that the purported mFLE may not be robust and warrants further scrutiny.

As for cognitive disfluency, we again found a robust effect of language on processing time: both decision and redecision times were significantly longer in the foreign language, reaffirming that the task was more cognitively taxing in a foreign language. However, and in contrast to Study 1, although the patterns mirror that of Study 1, FORs and FJCs are lower in the foreign language than native language, these differences did not reach statistical significance. Yet, relative proficiency (RP) emerged as a significant predictor: lower proficiency of the FL (vs. NL) was associated with lower FORs and FJCs. No effect of language or proficiency was found for decision reversals. Whereas, we observed consistent correlations between FOR and both decision time, redecision time, and the likelihood of decision reversals, underscoring the stability of this core meta-reasoning mechanism.

The null effect of language on FORs and FJCs may stem from several factors. In contrast to Study 1, participants in the foreign language group reported significantly higher FL proficiency and exposure compared to those in the native language group. This means that the sequential bilinguals in the foreign language condition, due to their high FL proficiency, could have reasoned more like they would have in their native language compared to those assigned in the native language condition. It is difficult to pinpoint the exact reason why the participants’ in the FL condition reported higher FL proficiency and exposure. A possible explanation could be that since participants had to pass a comprehension text in the language of the condition before being allowed to continue the experiment, therefore only those that were highly proficient continued on in the FL condition, though the root of this between group imbalance is beyond the scope of this research. Nevertheless, consistent with the modulatory effect of proficiency on the FLE (Del Maschio et al., 2022; Stankovic et al., 2022), we did observe similar trends in our measures of metacognitive monitoring. Thus, this null results for the main effect of language does not, however, invalidate the metacognitive hypothesis. Instead, the significant effects of RP point to a more nuanced mechanism. It appears it is not the binary status of a language as “foreign” that universally impacts metacognition, but rather the degree of cognitive strain an individual experiences while reasoning in it. RP serves as a proxy for this strain. For individuals with a large proficiency gap (high RP), the cognitive load is substantial enough to lower retrospective confidence. Whereas for highly proficient bilinguals, this effect is attenuated to the point of null significance. In summary, although our results of Study 2 do not support a statutory FLE on metacognition due to unmatching sequential bilinguals in terms of FL proficiency and exposure, we did reveal a more nuanced understanding of the such effect in relation to relative cognitive strain. While RP proved to be a powerful predictor, research could benefit from incorporating objective measures of language ability and more comparable samples to establish a more precise link between linguistic competence and metacognition.

Furthermore, despite previous research questioning the validity of the dilemmas used in the Process Dissociation approach (PDA) they have some utility in capturing FLE in moral inclinations. However, when combined with the intensive decision-redecision paradigm, they could have altered the decisional dynamics between System 1 and System 2, posing several limitations for the emergence of the FLE in metacognitive monitoring and control. Notably in Study 2 with DPA, each dilemmic scenario appears twice with the differences only in outcome, meaning that each participant saw each similar scenario 4 times during the experiment. Since repeated stimuli are processed more fluently (Reber & Schwarz, 1999; Whittlesea, 1993), it is possible that in increased processing fluency of the scenarios contributed to general higher levels of confidence (Thompson et al., 2011), thus clouding the emergence of the FLE in metacognition. Another limitation could be due to discrepancy in the nature of the stimuli. Whereas in Study 1, we contrasted the emotionally less evocative impersonal dilemmas and more evocative personal dilemmas, the stimuli in Study 2 do not much such distinction and are more abstract and structurally systematic by design. Since emotional content, or more broadly, affective states and arousal, significantly impact metacognition (Geurten & Lemaire, 2023; Massoni, 2014), it is plausible that the FLE on metacognition could be more pronounced in dilemmas that are affect-laden (i.e., personal dilemmas used in Study 1). Thus, the different sets of moral dilemmas used Study 1 and Study 2 could have explained the differential results, and this could suggest that the FLE on metacognition could be a combinatorial effect of the cognitive disfluency and emotional resonance inherent in thinking in a foreign language. In summary, findings of Study 2 should be considered in light of its limitations. First, while the PDA offers a more nuanced view than binary-choice models, it rests on the assumption that deontological and utilitarian processes are independent, a point of ongoing theoretical debate (Kunnari et al., 2020). Second, the repetitive and analysis-driven scenarios combined with the decision-redecision paradigm may not be suitable to effectively detection the FLE in metacognition.

Synthesizing the two studies, a clearer picture emerges. The most stable and robust consequence of foreign language use in moral reasoning is an increase in processing time, a direct index of increased cognitive disfluency. The core architecture of meta-reasoning also remains stable: a low FOR consistently predicts greater analytical engagement. The critical variable is what triggers the drop in FOR. In highly charged, emotional contexts (Study 1), the foreign language itself acts as a potent cue for uncertainty. In more sterile, analytical contexts (Study 2), the effect is more subtle, emerging only for those individuals for whom the foreign language poses a significant cognitive challenge.

## 6. General Conclusions

This research investigated the mFLE from a metacognitive perspective. Across two pre-registered studies, we sought to determine whether the cognitive and affective differences associated with foreign language could alter metacognition. While our findings did not replicate the oft-cited effect of a foreign language on moral decisions and moral inclinations, they provide compelling yet nuanced evidence for a FLE in metacognition.

The central takeaway from this research is that the FLE’s influence may not be reliably detected in decision outcomes, but rather in the cognitive and metacognitive processes taken to reach them. Across two distinct experimental paradigms, the use of a foreign language consistently increased cognitive disfluency, as evidenced by significantly longer decision times. This finding forms the bedrock of our metacognitive model: a foreign language context introduces cognitive disfluency. This disfluency, in turn, can serve as a potent metacognitive cue, lowering the initial Feeling of Rightness (FOR). As our results repeatedly demonstrated, a lower FOR acts as a reliable metacognitive signal in predicting greater subsequent allocation of cognitive resources—manifesting as longer re-evaluation times and, in certain contexts, a higher likelihood of reversing an initial decision. This metacognitive account provides a parsimonious explanation for why the mFLE is so often elusive at the behavioral level, as the engagement of more deliberate, System 2 processing does not guarantee a change in the final outcome, but it may serve to scrutinize and ultimately reaffirm the initial intuition, akin to acquiescence (cf. Hu et al., 2024). Our findings suggest that the FLE on metacognitive monitoring is the more fundamental phenomenon, as the impact of this monitoring on final choice appears to be conditional, dependent on factors such as the affective intensity of the context (as evidenced by the differing results of Study 1 and Study 2) and the individual’s cognitive strain, for which relative proficiency serves as a powerful proxy. This metacognitive account does not necessarily supplant existing theories but rather integrates them. The “reduced emotion” and “increased deliberation” hypotheses can be elegantly reconciled within this account. Reduced emotional resonance and the increased cognitive effort of language processing are not mutually exclusive; both can be conceptualized as sources that diminish metacognitive ability.

The implications of this research extend beyond the FLE itself. For decision science, our findings issue a strong call to look beyond final outcomes and incorporate process-level variables. Measures of cognitive and metacognitive processes are not merely epiphenomenal but are critical windows into the underlying cognitive architecture. For our increasingly globalized and multilingual world, the findings carry practical significance. In fields from international diplomacy and business negotiations to legal proceedings involving non-native speakers, our work suggests that language context matters. Even if a non-native speaker arrives at the same conclusion as a native speaker, their metacognitive experience may be markedly different—characterized by greater uncertainty and more cautious, deliberate processing. Recognizing this can foster more equitable and effective communication in high-stakes cross-linguistic environments.

The conclusions of this registered report must be tempered by an acknowledgment of its limitations. Our investigation was confined to an online sample of a single language pair (viz., English-Spanish) and a specific national group (viz., United States), which may limit generalizability. Furthermore, the use of hypothetical moral dilemmas, a standard in the field, may not fully capture the complexities of real-world moral behavior. These limitations illuminate clear pathways for future research. This meta-reasoning paradigm should be extended to different language pairs, particularly those with greater linguistic distance, and to diverse participant populations to test the universality of the proposed mechanisms. Employing more direct, physiological measures of fluency and affect could provide convergent evidence. Finally, applying this metacognitive framework to other domains of judgment where the FLE has been observed, such as risk assessment and classic framing effects, would be a fruitful endeavor. Such work will be essential to building a comprehensive and robust theory of how the languages we speak and see influence the way we feel and reason.

## Data Accessibility Statement

All stimuli, raw data, analyses, and supplementary materials are openly available in the OSF repository (https://osf.io/m7kc8).

## Footnotes

We acknowledge the suggestion by an anonymous reviewer for the inclusion of Study 2 during Stage 1 reviewing process of this registered report.

Due to a collective error in the Stage 1 registered report, mixed effects models are not suitable for analyzing U and D scores as each participant is associated with a unique score. Thus, the results for U and D scores were analyzed with general linear models.

Although we had preregistered poisson distribution for the analysis of Decision Reversals, however, this distribution was not suitable for analyzing whether a decision is reversed or not. Thus, the results for Decision Reversals were analyzed with a binomial distribution.

Although we had preregistered the Spearman method for the analysis of the relationships between FOR and DT, RDT, and DR, however, the Spearman method was not suitable for these analyses. Thus, the results for these correlations were analyzed with generalized linear models (GLMs), consistent with the analytical strategies in this paper.
